# Supplementary material for: Low-cost measurement of face mask efficacy for filtering expelled droplets during speech
Source: Sci Adv. 2020 Sep 2;6(36):eabd3083. doi: 10.1126/sciadv.abd3083 (PMC7467698; doi:10.1126/sciadv.abd3083)
Supplement: abd3083_SM.pdf [file abd3083_SM.pdf]

Science Advances
AAAS

advances.sciencemag.org/cgi/content/full/sciadv.abd3083/DC1

Supplementary Materials for

Low-cost measurement of facemask efficacy for filtering expelled droplets during speech

Emma P. Fischer, Martin C. Fischer*, David Grass, Isaac Henrion, Warren S. Warren, and Eric Westman

*Corresponding author. Email: martin.fischer@duke.edu

Published 7 August 2020, *Sci. Adv.* **6**, eabd3083 (2020)
DOI: 10.1126/sciadv.abd3083

This PDF file includes:

Materials and Methods
Figs. S1 to S7

Supplementary Materials

Materials and Methods

Camera specifications and acceptance angle

We used a cell phone camera (Samsung Galaxy S9) for recording the videos. It was mounted at a distance of 20 cm from the light sheet. Using the Android app "Open Camera" the frame size was set to 1920 x 1080 pixels, the focal distance to 20 cm, the exposure time to 1/50 s, and the frame rate to 30/s. At this focal distance, each camera pixel recorded an area of 120 µm x 120 µm at the position of the light sheet. The camera was operated with an opening aperture setting of 2.86mm. Therefore, the camera covered a polar and an azimuthal angle of $\theta_0 = \phi_0 \approx 1°$ with respect to the light sheet. Note that the polar angle θ_0 indicated in Fig. 4 (B) is exaggerated for better visualization.

Camera calibration and sensitivity

We were unable to find detailed specifications on the camera sensitivity and therefore calibrated it ourselves. In brief, we attenuated a laser pointer with neutral density filters and shined it onto the camera while recording a short (~1 s) video with the same recording settings used during the experiment. In Fig. S7 we show the laser spot recorded with the camera for eight different laser powers (17 fW to 240 fW). The pixel values represent the maximum value over all frames. Interestingly, while the maximum values are in the 30- to 40-count range (out of the maximum of 255 for the 8-bit camera) for the lowest power, the mean over all frames is almost 0. We attribute this discrepancy to the lossy compression algorithm used during video encoding. For regions with pixel counts over the 30- to 40-count range, this effect decreases strongly.

We compute the integrated pixel count in the region of interest as a function of laser power (=energy deposited on the camera) and find a linear relationship with a slope of $m \approx 1.5 \times 10^{18} \frac{counts}{J}$ (counts per Joule). Assuming a camera 'threshold' of 40 counts, a minimum energy of $E \approx 2.3 \times 10^{-17} J \approx 75$ photons is required to generate a camera signal. This corresponds to a laser power of $P = 1.1\, fW$ over the exposure time of a single frame (1/50 s). Note that this sensitivity holds only for ideal conditions in which there is no noise, and all photons impinge on the same pixel. If the photons are distributed over a larger area, like in Fig. S7 for example, more photons are required to generate a signal. For this reason, we declare all signals below a threshold of 70 counts as noise and only use pixels with more than 70 counts for droplet detection.

Background and edge count removal

A weak background is present in the video recordings, which is predominantly located in a horizontal stripe across the field of view and at the left and right edge of the field of view, as can be seen in Fig. S6. An image that contains only this weak background signal is derived by averaging all frames of a video to a single frame and simultaneously setting all values above a noise threshold value to 0. This effectively "erases" all droplets from the video because their pixel values are above the threshold. The introduced discontinuity at a droplet position is negligible due to averaging over all frames, resulting in a smooth background image. This background image is subtracted from each frame of the same video. The resulting images are binarized with a threshold value t_{bin}. This means that all pixels with a value above t_{bin} are set to 1 (as well as surrounding connected pixels) and all pixel values below the threshold are set to 0. We chose a value of $t_{bin} = 70$ (out of 255 for an 8-bit image) as a compromise between detecting small droplets, which scatter few photons and consequently have a low pixel value, and efficient suppression of background

and noise. During the calibration, we found that the video encoding suppresses weak signals below 30-40 counts (out of 255), as described above. As we want to avoid false detection events, we consider the range below 70 as noise and only declare a value above 70 as detected droplet. A pixel value of 70 corresponds to a minimum of 132 detected photons and this lower limit defines the sensitivity of our measurement. From Fig. 4 (B) we find that this corresponds to a droplet size of 0.15 µm.

When all droplet positions are plotted in a two-dimensional histogram, they map the spatial mode of the illuminating Gaussian laser beam. Sometimes, the algorithm returns droplets in regions without laser light and in regions very close to the left and right edge of the video, which is noise wrongfully classified as droplets. We remove those false detection events by rejecting every droplet that is more than two beam diameters away from the laser beam and by removing droplets that are in the direct vicinity of the left or right edge.

Histogram generation

The data evaluation algorithm returns droplet trajectories with a measurement of minor and major axis in each frame. A visual representation is shown in the inset of Fig. S6. The image is a projection of 10 consecutive frames and the droplets are highlighted with a colored best-fit ellipsoid (the plotted ellipsoid is enlarged for better visualization) and their corresponding frame number. Same colors belong to the same trajectory. We use the maximum value of the minor axis of each trajectory and generate a histogram for each face mask. The histograms are shown in Fig. S5; each face mask (orange color) is compared to the control trial (no mask) which is plotted in green color. We want to stress that the x-axis of the histogram “binary diameter” does not represent a direct measurement of the droplet size. It is merely a qualitative measure that can be used for relative comparison, because the resolution of the camera is 0.12 mm/pixel, which means the smallest droplet size that can be imaged is limited by this resolution. However, droplets below the resolution limit are still recognized if the particle scatters enough light into the camera (see section on Camera Calibration and Sensitivity). This means that we can make qualitative statements about the droplet sizes, for example in the neck fleece case. The neck fleece has a higher total droplet count compared to the control trial. When comparing the histogram, it appears that the fleece transmits fewer large droplets (above the resolution limit) and more small droplets (below the resolution limit). We interpret this as the neck fleece dispersing large droplets into several smaller droplets.

Supplementary figures

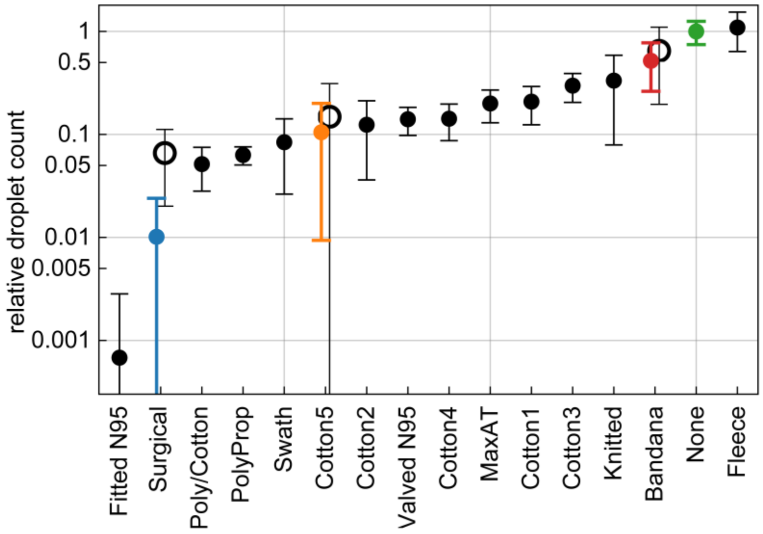

Fig. S1. Droplet transmission through face masks, displayed on a logarithmic scale. Relative droplet transmission through the corresponding mask. Each solid data point represents the mean and standard deviation over 10 trials for the same mask, normalized to the control trial (no mask), and tested by one speaker. The hollow data points are the mean and standard deviations of the relative counts over four speakers.

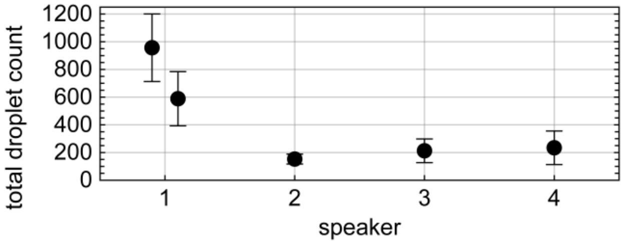

Fig. S2. Total droplet count without mask for all speakers. The points and error bars represent the mean value and distribution standard deviation, respectively, of the total droplet count without mask. Speaker 1 has two entries: one for the data set testing all the masks, the other as part of the four-speaker set testing the subset of masks.

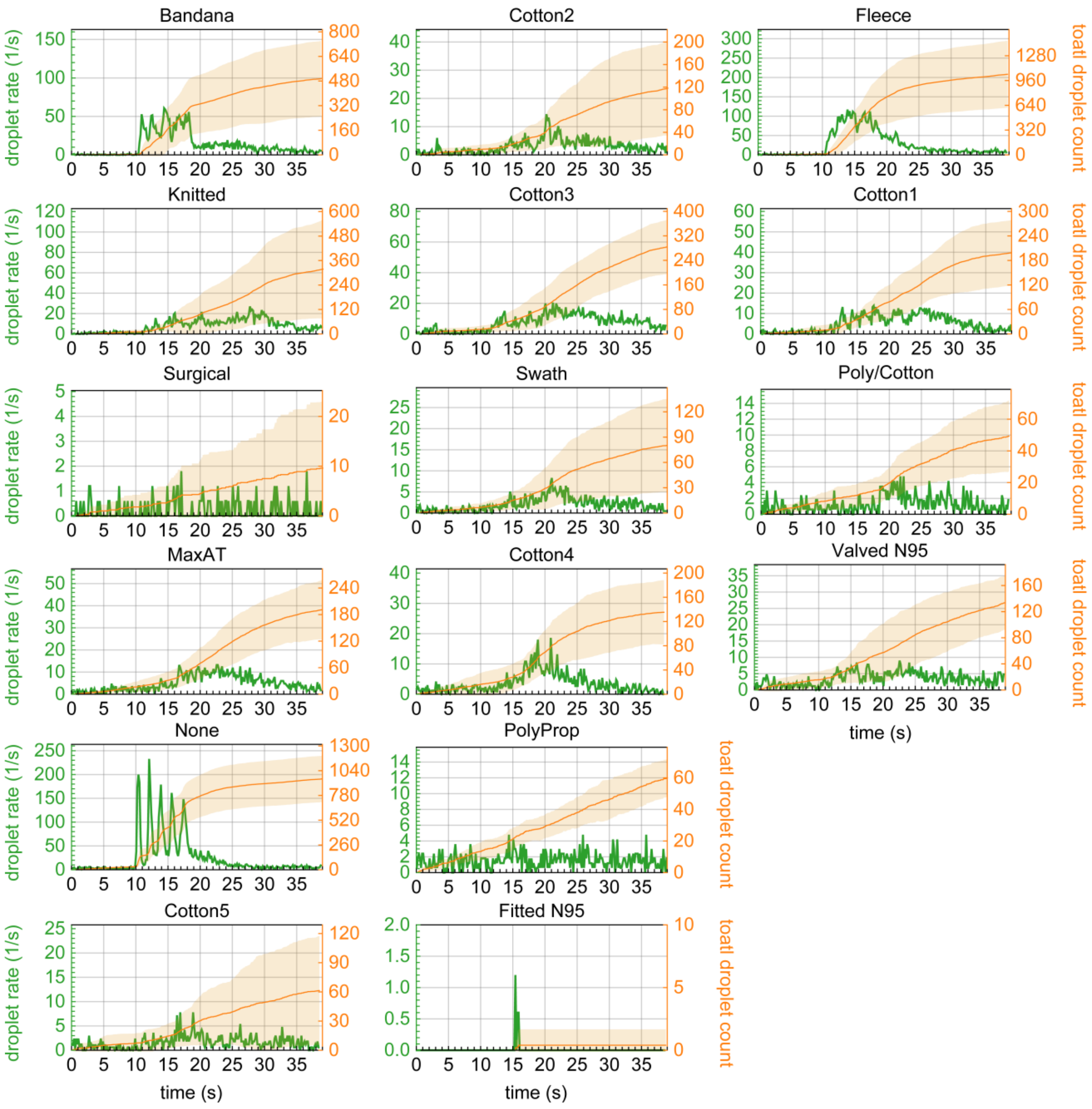

Fig. S3. Time evolution of droplet rate and total droplet count for one speaker. The green curve shows the droplet rate as a function of time (left axis), averaged over all repetitions, for each face mask. The solid orange line represents the total droplet count as a function of time (right axis), averaged over all repetitions, for each face mask. The orange shaded area indicates one standard deviation.

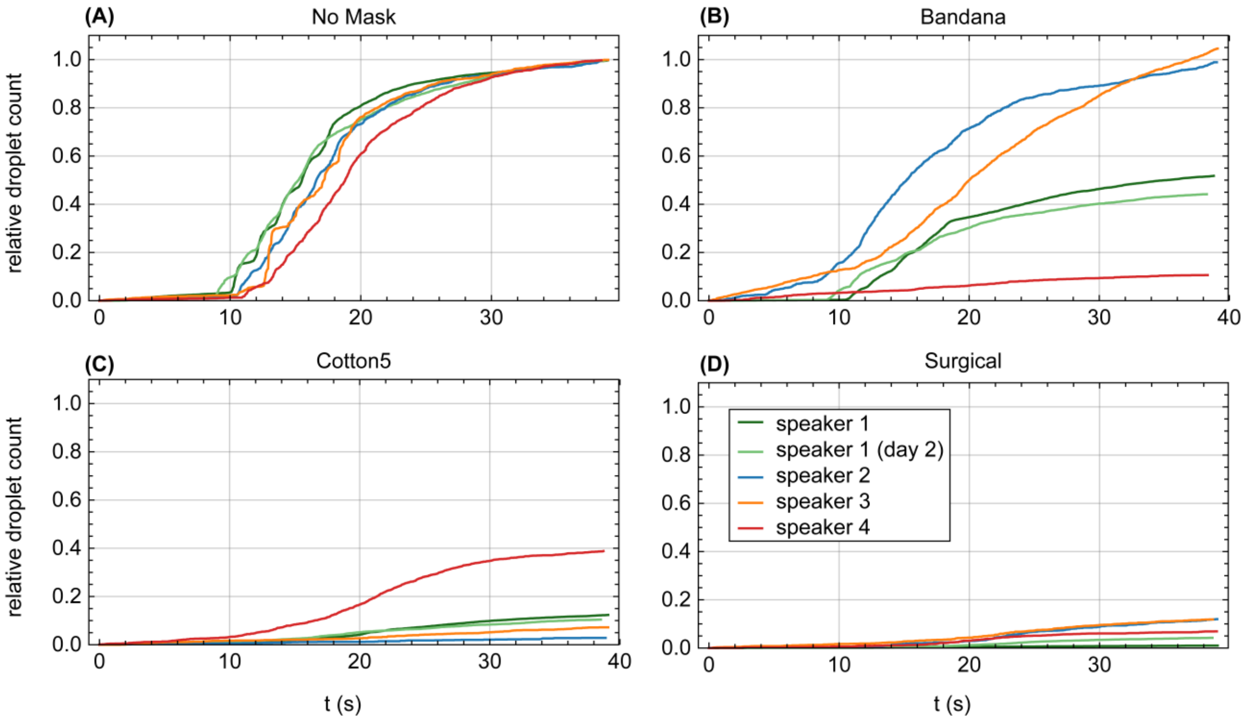

Fig. S4. Time evolution of the relative droplet count for all speakers. The lines represent the relative droplet count as a function of time, averaged over all repetitions, for each speaker. Speaker 1 has two entries: one for the data set testing all the masks, the other as part of the four-speaker set testing the subset of masks (day 2).

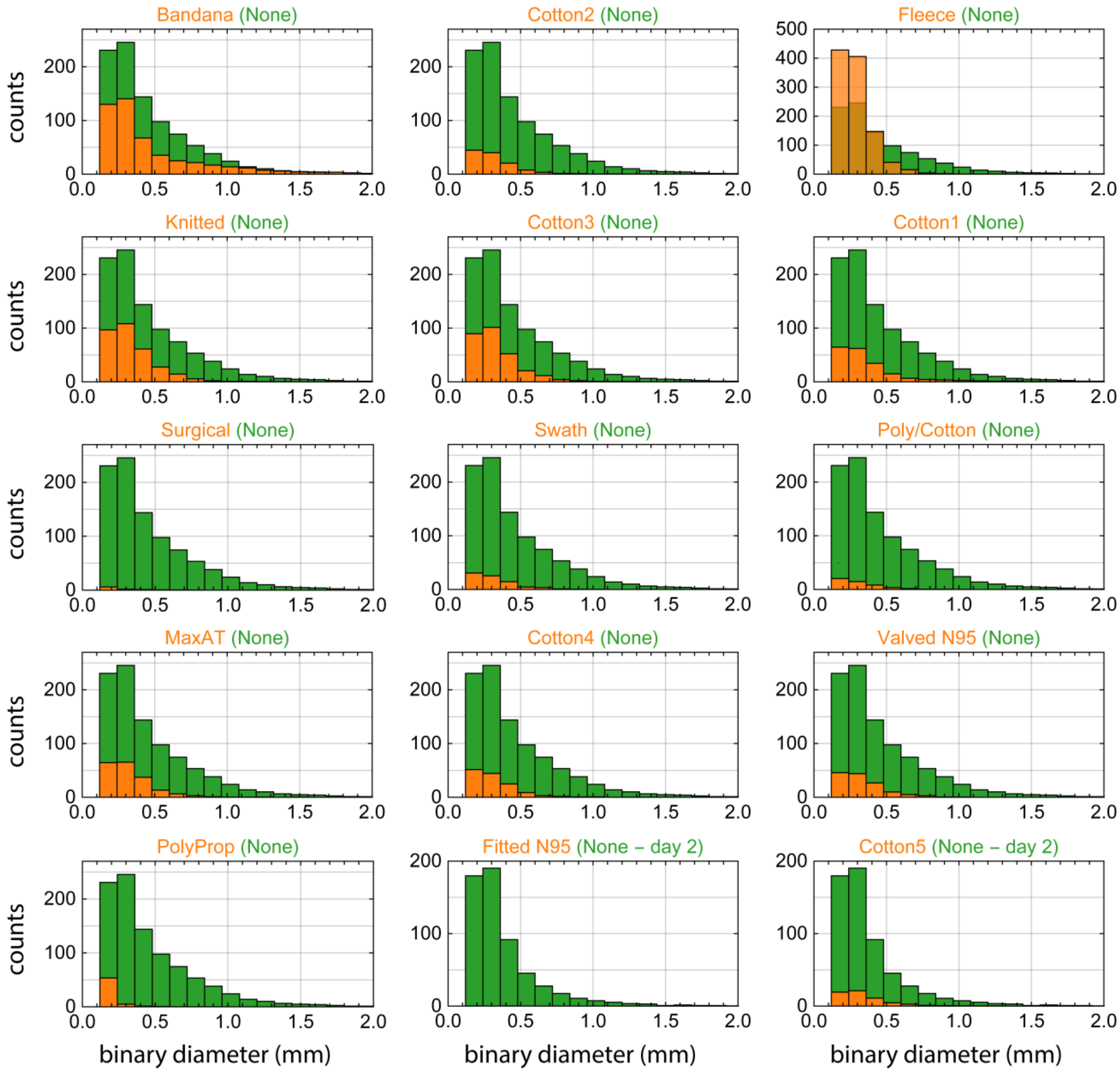

Fig. S5. Qualitative size histogram. Note that the size of droplets smaller than the pixel resolution (120 µm) cannot be reliably measured. Also, note the decrease of large and the increase of smaller particles numbers for the neck fleece. We attribute this to the breakup of large droplets into several smaller ones when passing through the material. The measurements “Fitted N95” and “Cotton5” were performed by the same speaker as all other measurements, but on a different day. Therefore, they have a different control trial histogram (None – day2).

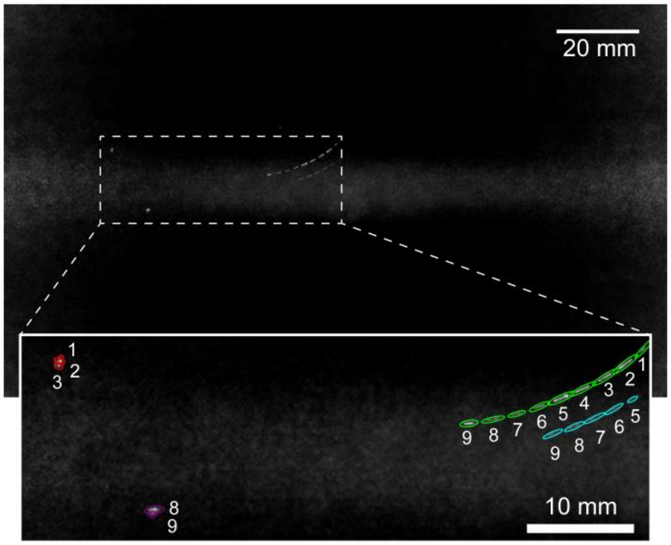

Fig. S6. Example frame projection demonstrating particle trajectories. The image shows the maximum intensity projection of 10 consecutive frames for the entire field of view. The horizontal stripe in the middle as well as the area on the left and the right edge of the image are background signals from diffuse laser reflections, not scattered light from particles. The inset highlights four droplet trajectories that are color coded (one color per droplet) and labeled with the frame number.

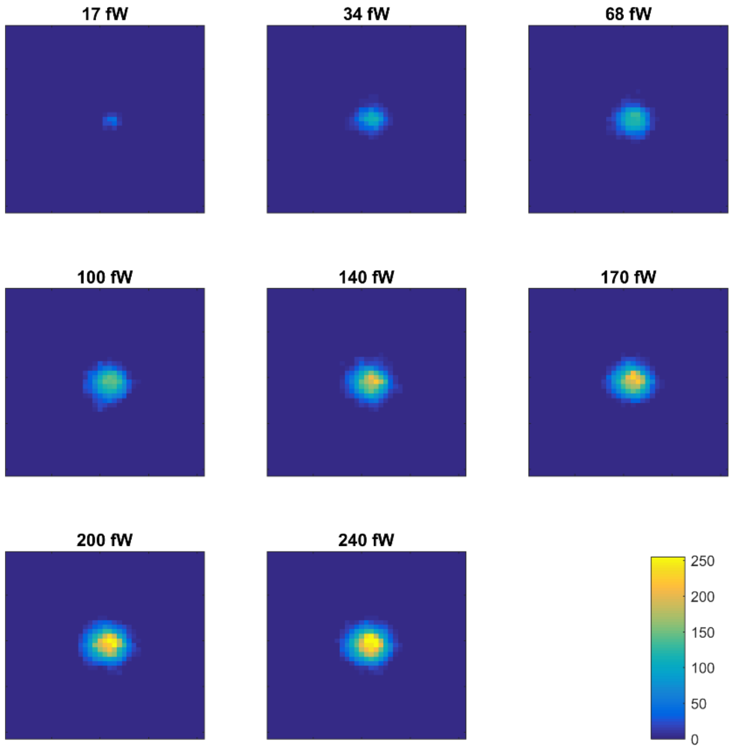

Fig. S7. Camera calibration and sensitivity. An attenuated laser beam of beam powers indicated in the figure illuminated the camera during a short video. The images show the illuminated region of the camera (40 x 40 pixels) as a maximum intensity projection over all frames. Note that the laser beam is not focused on a single pixel but was deliberately defocused.
